# Potential use of insect protein and phytonutrient-based tropical plant supplementation on rumen fermentation characteristics and microbial protein synthesis in Thai native beef cattle

**DOI:** 10.5713/ab.25.0166

**Published:** 2025-06-24

**Authors:** Burarat Phesatcha, Kampanat Phesatcha, Maharach Matra, Thiwakorn Ampapon, Metha Wanapat

**Affiliations:** 1 Department of Applied Biology, Faculty of Science and Liberal Arts, Rajamangala University of Technology Isan, Nakhon Ratchasima, Thailand; 2 Department of Animal Science, Faculty of Agriculture and Technology, Nakhon Phanom University, Nakhon Phanom, Thailand; 3 Division of Animal Science, Department of Agricultural Technology, Faculty of Technology, Mahasarakham University, Maha Sarakham, Thailand; 4 Department of Animal Science, Faculty of Agriculture and Technology, Rajamangala University of Technology Isan, Surin Campus, Surin, Thailand; 5 Tropical Feed Resources Research and Development Center (TROFREC), Department of Animal Science, Faculty of Agriculture, Khon Kaen University, Khon Kaen, Thailand

**Keywords:** Digestibility, *Gryllus bimaculatus*, Phytonutrient, Rumen Fermentation, Ruminants

## Abstract

**Objective:**

This experiment evaluated the use of insect protein and phytonutrient-based tropical plant supplementation on rumen fermentation characteristics and microbial protein synthesis in Thai native beef cattle. Enhancing protein utilization and promoting rumen fermentation could be achieved by combining high-protein crickets and phytonutrients from mangosteen peel and lemongrass powder to a pellet (CMLP).

**Methods:**

Four native male Thai beef cattle were randomly assigned treatments using a 4×4 Latin square design to receive four dietary treatments. The treatments were as follows: control (no supplementation), CMLP supplement at 50 g/h/d, CMLP supplement at 100 g/h/d and CMLP supplement at 150 g/h/d, respectively.

**Results:**

Results revealed that the supplementation of CMLP in beef cattle did not influence rice straw intake, concentrate intake and total feed intake, which also enhanced the digestibility of crude protein, acid detergent fiber and neutral detergent fiber (p<0.05). Additionally, volatile fatty acid production of propionate and bacterial population were increased (p<0.05), and protozoal populations and production of methane decreased (p<0.05) with a higher level of CMLP supplementation. Furthermore, efficiency of microbial nitrogen synthesis significantly increased by increasing the level of CMLP supplementation, particularly at 150 g.

**Conclusion:**

Our results suggested that CMLP supplementation, particularly at 150 g/h/d, enhanced nutrient digestibility, increased the propionic acid proportion, and promoted microbial protein synthesis while reducing protozoal populations and methane production. CMLP showed promise as an effective dietary protein supplement that improved rumen fermentation and performance of Thai native beef cattle.

## INTRODUCTION

Many obstacles in modern livestock production impact the development and procreation of animals to provide meat, milk, and skin. Animal protein demand continues to increase globally, generating a greater need to investigate sustainable alternatives to traditional sources of livestock feed. One promising solution is integrating insects into animal feed [1]. Prices of traditional protein sources have spiked owing to yield variables and competition between animals and people, making protein the most expensive ingredient in feed compositions [2]. Future animal production systems will need to identify new sources of high-quality and sustainable protein ingredients to satisfy the need for highly nutritious animal feed. Insects are a viable alternative feed source containing elevated protein and fat contents with excellent feed conversion rates [3]. Insect proteins can be used to substitute conventional soy and fish meal in animal feed formulations [4]. Various insect species such as crickets, grasshoppers, mealworms, locusts, black soldier fly larvae, and house fly maggots have been examined for their viability as alternatives to soybean meal in domestic animal feed and their digestibility, fermentation profiles, and methane production in the rumen [5]. *Gryllus bimaculatus*, a species of cricket within the Gryllinae subfamily, is characterized by high crude protein (CP) content exceeding 60%–70% of dry matter (DM), along with a favorable amino acid profile including lysine, threonine, and tryptophan [6]. This insect contain 20% fat, while *Acheta domesticus* has a lipid composition with abundant palmitic, oleic, and linoleic acids [7]. Palmquist and Jenkins [8], reported that ruminal microorganisms including cellulolytic bacteria and protozoa which break down carbohydrates are negatively affected by high-fat feedstuffs that limit the cellulolysis of structural carbohydrates. Changes in the protein concentration and fatty acid (FA) composition of insects can impact the process of ruminal breakdown [9]. The rumen fermentation characteristics and nutrient digestibility were not negatively affected when four edible insects such as the house cricket, the Taiwan giant cricket, the two-spotted cricket, and the silkworm were used to replace 25% of soybean meal in a 60:40 forage-to-concentrate diet [10]. Moreover, Phesatcha et al [11] demonstrated that Thai native beef cattle had better fermentation and digestibility when cricket meal pellets were used instead of soybean meal while Astuti et al [12] suggested that rumen fermentation profiles of post-weaning goat kids were unaffected when 30% cricket meal was utilized as a substitute for soybean meal in the feed. Phytogenic compounds, which include plant-based nutrients like saponins (SP) and condensed tannins (CT), improve animal well-being by lowering ruminant methane emissions and increasing bovine production [13]. Tannins are polyphenolic chemicals that exhibit a strong affinity for proteins. When exposed to typical ruminal conditions, these active ingredients form stable rumen complexes that prevent the degradation of dietary protein [14]. Matra and Wanapat [15] showed that tropical plants and fruit peels can enhance rumen fermentation as feed additives, hence benefiting ruminants. Lemongrass (*Cymbopogon citratus*) is a herbal plant containing the essential oil citronella that has shown positive effects in beef cattle on protein synthesis, rumen ecology digestibility, and microbial populations [16]. Results indicate that lemongrass benefits animal production, ruminal fermentation, antioxidant activity, FA composition, and total bacterial population without negatively impacting ruminant health [17]. The peel of mangosteen (*Garcinia mangostana*), an agricultural by-product from tropical regions, contains 16.7% crude tannins (CT) and 9.8% SP, indicating its potential for use in ruminant feed [18]. Additionally, swamp buffaloes given 100 g/head/day of mangosteen peel supplementation showed reduced methanogen populations with enhanced microbial protein production [19]. Enhancing protein utilization and promoting rumen fermentation could be achieved by combining high-protein crickets and phytonutrients from mangosteen peel and lemongrass powder. However, scant research has been conducted to assess the effects of insect protein and phytonutrient supplementation on nutritional digestibility, rumen fermentation, and microbial protein synthesis. This investigation evaluated insect protein and phytonutrient based tropical plant (CMLP) feed supplementation on rumen fermentation characteristics, microbial protein synthesis and methane emission in Thai native beef cattle.

## MATERIALS AND METHODS

### Ethical procedure

This study was approved by the Animal Care and Use Committee of Rajamangala University of Technology Isan, Thailand (approval no. 01-67-003). According to Thailand’s National Research Council’s Ethics of Animal Experimentation, approval was required to collect rumen fluid from animals for this study’s main objective, which comprised laboratory examination of ruminant feeds.

### Preparation of insect protein and phytonutrient pellets

Forty-day-old *Gryllus bimaculatus* adult field crickets were sourced from a cricket farm in Roi Et Province. The insects were dried in a hot air oven at 60°C. Phytonutrient-based tropical plant contain mangosteen peel powder and lemongrass powder used in the experiment. Cricket meal, mangosteen peel powder and lemongrass powder were ground to a particle size of 1 mm (Cyclotech Mill; Foss Tecator AB). Pellet (CMLP) was prepared by combining 50% dried cricket powder, 20% mangosteen peel powder, 20% lemongrass powder, 9% cassava chips, and 1% molasses. A pellet machine was used to carry out the pelleting procedure (Victor Pellet Mill). The pellets were sun-dried to attain 90% DM before animal consumption.

### Dietary treatments and experimental design

Four Thai native male beef cattle with starting body weight (BW) 220±10 kg were randomly assigned to four treatments using a 4×4 Latin square design without CMLP (Control), CMLP supplementation at 50 g/h/d, CMLP supplementation at 100 g/h/d, and CMLP supplementation at 150 g/h/d. A concentrate of 1.0% BW was given to all animals, supplemented with CMLP at the given treatments. The animals received two equal meals of rice straw (fed *ad libitum*) each day at 07:00 and 16:00 hours. Dietary treatments were given to the animals in four separate sessions, with each session lasting 21 days. During each session, the first 14 days were used for adaption to the treatment, with samples collected and determination of the digestibility of the nutrients during the final 7 days. Table 1 presents a summary of the chemical compositions and constituents of both the diets and the CMLP.

### Sample collection and chemical analyses

The offered and refused feeds were weighed before the morning feeding to measure the daily individual feed intake of concentrate, rice straw, and CMLP. Samples of feed, feces, and urine were collected for analysis during the final seven days of each session. Following oven-drying, composite samples were ground to a particle size of 1 mm and then examined for DM, ash, CP [20] and acid-insoluble ash [21]. Acid detergent fiber (ADF) and neutral detergent fiber (NDF) as described by Van Soest et al [22]. The CMLP samples were analyzed for CT concentration according to the methodology established by Wanapat et al [23]. Feces and urine samples were collected during the last seven days of each period. The feces were collected by rectal sampling, while urine sample was collected by spot sampling following, manual stimulation of the penis to stimulate urination. The concentrations of allantoin and creatinine in the urine were measured using high performance liquid chromatography (HPLC) following the methodology of Chen and Gomes [24], while the quantity of ingested microbial purines was assessed based on the excretion of purine derivatives. The microbial crude protein (MCP) was ascertained using the methodology of Galo et al [25]. Rumen fluid and jugular blood samples were taken at 0 and 4 hours post-feeding on the final day of each sampling session. Approximately 200 cc of rumen fluid was extracted from the rumen using a stomach tube linked to a vacuum pump. The temperature and pH of the rumen fluid were promptly assessed using a portable pH meter (Hanna Instrument HI8424 Microcomputer). The rumen fluid samples were filtered twice through four layers of cheesecloth and then separated into two equal portions. The first portion was subjected to ammonia nitrogen (NH_3_-N) and volatile fatty acid (VFA) analysis by adding 5 mL of 1M H_2_SO_4_ to 45 mL of rumen fluid and centrifuging at 1,600×g for 15 minutes. The NH_3_-N was calculated using the micro-Kjeldahl technique [20], and the concentrations of rumen VFA profiles were analyzed using HPLC [26]. Methane (CH_4_) production in the rumen was indirectly estimated based on the concentrations of VFAs by using the methods of Moss et al [27]. For the second portion, a hemocytometer was used to conduct a total direct counting of the populations of bacteria and protozoa following Galyean [28]. At 0 and 4 hours after feeding, blood samples (5 mL) were collected from the jugular vein, mixed with ethylenediaminetetraacetic acid (EDTA), and analyzed for blood urea nitrogen (BUN) [29].

### Statistical analysis

The results were analyzed as a 4×4 Latin square design using the General Linear Model (GLM) procedure of SAS software [30]. Significant differences among treatment means were analyzed according to Duncan’s New Multiple Range Test [31], with a p-value set at less than 0.05. Orthogonal polynomial contrasts were used for statistical comparison of the treatment trends.

## RESULTS AND DISCUSSION

### Chemical composition of experimental feeds

The ingredients and chemical content of the diets are shown in Table 1. The concentrate included 14.7% CP, while rice straw (2.3% CP, 75.5% NDF, and 47.4% ADF) was fed as a roughage source. The chemical composition of CMLP contained 38.5% CP, 27.2% NDF, and 18.7% ADF. CMLP comprised phytonutrient compounds, particularly 8.6% CT. Taufek et al [32] showed that crickets contained a higher concentration of CPs, exceeding 50%, compared to other insects. The CMLP had a high concentration of protein as well as favorable physical properties and was readily ingested by the animals. Cricket meal pellets contained 62.4% CP, 6.9% CF, and 14.7% EE according to Phesatcha et al [11]. Jayanegara et al [5] reported that crickets have high raw protein content at above 40% DM. Tannins are polyphenolic substances that exhibit a strong affinity for proteins. In typical ruminal conditions, these active ingredients form stable rumen complexes that prevent the degradation of dietary protein [14].

### Feed intake and nutrient digestibilities

The feed intake and nutrient digestibility are presented in Table 2. Rice straw intake, concentrate intake, and total feed intake were not impacted by CMLP supplementation in beef cattle (p>0.05). The addition of CMLP increased the digestibility of CP, while the digestibility of NDF and ADF reduced (p<0.05). Addition of CMLP did not change the digestibility of DM and OM (p>0.05). The level of CMLP supplementation did not influence DM consumption because CMLP did not negatively impact diet palatability. The cricket, rich in protein and fat enhanced ruminant nutrition. The residues of crickets (*Acheta domesticus*) and mealworms (*Tenebrio molitor*) exhibit high digestibility and low carbohydrate profiles. They are devoid of preservatives, antibiotics, hormones, and pesticides [33]. Consistent with our findings, Phesatcha et al [11] showed that utilizing insects with higher quality and varied amino acid profiles as an alternative to traditional protein sources enhanced beef growth performance. Apri and Komalasari [34] reported that substituting soybean meal with varying percentages of cricket meal up to 30% did not adversely impact rumen fermentation profiles or result in significant differences in daily weight growth. The dietary CP content in animal diets is essential for promoting growth. Improving the level of CMLP supplements in the diet enhanced nutritional digestibility but exerted little influence on the digestibility of DM and OM. Dung et al [35] concluded that using a high CP concentrate enhanced CP digestibility but did not influence DM, OM, and NDF digestibilities. The addition of CMLP enhanced CP digestibility because of the interaction between CT and protein which facilitated an increase in rumen by-pass protein. True protein, also known as bypass protein, and MCP are important to ruminants. The pelleting process protects amino acid digestion by rumen bacteria. Phytogenic chemicals influence the rumen microbiota, which affects fiber digestion, methane production, and the biohydrogenation of unsaturated FAs. Mangosteen peel and lemongrass powder possess high concentrations of tannins, which have been shown to inhibit protein degradation in the rumen through amino bypass synthesis [19]. In this case, a decrease in rumen protein digestion is beneficial because it dissociation of tannin-CP bonds, which allows lower gut digestion of proteins. Moreover, Matra and Wanapat [15] found that the addition of dragon fruit peel pellet enhanced the digestibility of CP in dairy cows.

### Rumen fermentation characteristics, blood metabolites and microbial populations

Different CMLP supplementations had no significant effects on ruminal temperature, pH, and BUN concentration (p> 0.05), as shown in Table 3. The pH values ranged from 6.5 to 6.8 for all treatments and facilitated the activities of microorganisms, particularly cellulolytic bacteria, during rumen fermentation. Van Soest [36] indicated a pH range of 6.2 to 7.2 for ideal microbial activity. Strategic feedstuffs enriched with phytonutrients enhance rumen fermentation efficiency by sustaining elevated pH levels [37]. There were no significant changes in the concentration of ammonia nitrogen (NH_3_-N) as a result CMLP of supplementation, within the normative range of 15.2 to 17.0 mg/dL. Adding CMLP decreased the concentration of NH_3_-N because the tannin-protein combination facilitated the proteolysis process, limiting protein breakdown and diminishing NH_3_-N concentration. CTs demonstrate the important ability to bind CP in the rumen, hence diminishing dietary protein loss due to ammonia production and improving protein utilization [38]. Phesatcha et al [39], reported that CT positively affected nutrition by creating a protein-tannin complex, reducing the degradation of ruminal feed protein, and lowering NH_3_-N synthesis. The slow release of nitrogen was promoted by the specific formulation of the feed supplement in the form of pellets, with limited degradation in the rumen [40]. Our results indicated that BUN levels remained unchanged in animals consuming the CMLP group compared to the non-supplemented group, ranging from 11.0 to 12.5 mg/dL. Byers and Moxon [41] stated the optimal range for BUN at between 10.0 and 15.0 mg/dL. The production of NH_3_ in the rumen is closely correlated with BUN levels. Reduced BUN concentrations have been linked to the incorporation of CT and SP in the diet [42].

The propionate (C_3_) concentration increased, whereas acetate (C_2_) concentration decreased (p<0.05) at higher levels of CMLP supplementation. However, the supplementation of CMLP in cattle did not significantly affect total VFA, butyrate (C_4_), and the ratio of acetate to propionate (C_2_ to C_3_ ratio) (p>0.05). Findings showed that rumen methane production decreased and bacterial populations increased (p<0.05) at higher levels of CMLP supplementation, whereas protozoal populations decreased (p<0.05) with varied dosages of CMLP (Table 3). The efficacy of the microbial population and their functions in the rumen is contingent upon rumen fermentation, which facilitates the synthesis of VFAs, serving as the energy substrate for maintenance and growth, with propionic acid acting as a principal glycogenic precursor [43]. This process was ascribed to the reduction of acetate, leading to an increase in propionate while propionate remained unchanged. The propionate concentration often improves when rumen methanogenesis is suppressed, as demonstrated in this study. Our results indicated that using a tannin source alongside insects may enhance the concentration of propionate. Tannin’s biological role may include inhibiting the growth of acetogenic bacteria while altering the pathway by which H_2_ generates propionate. A reduced acetate to propionate ratio may be associated with elevated propionate concentration [44]. Phesatcha et al [45] showed that the addition of *Flemingia macrophylla* pellets increased the proportion of C3 while decreasing the proportion of C2 in beef cattle. Ahmed et al [46] and Apri and Komalasari [34] reported that substituting up to 25% of SBM with edible insects (*Gryllus bimaculatus, Acheta domesticus*, *Bombyx mori*, and *Brachytrupes portentosus*) and 30% with cricket meal had no negative impact on the rumen fermentation profile. Insect oils rich in 12:0 and 14:0 positively affect ruminal function by suppressing methane and ammonia generation, respectively [47]. Thus, rumen methane production reduced, leading to a subsequent reduction in protozoal numbers. As a result, phenolics and flavonoids derived from multifunctional tropical plants possess the capacity to directly suppress methanogen populations and their activity. Phesatcha et al [48] reported that tropical plants containing CT and SP such as *Garcinia mangostana* peel, caused a decrease in protozoan and methanogen populations and a reduction in CH_4_ emissions in cows. In addition, Ampapon et al [49] demonstrated that the addition of phytonutrient pellets reduced CH_4_ generation by inhibiting ruminal protozoa. Substituting soybean meal with cricket meal pellets enhanced propionate levels while reducing acetate levels and methane emissions in beef cattle [11]. Beyzi [50] found that plant essential oils affect rumen ecology by influencing the cell membranes of rumen bacteria and protozoa, hence decreasing hydrogen concentration and methanogenesis. The bacterial population increased with higher levels of CMLP pellets, whereas the protozoal population declined. The addition of cricket meal enhanced the bacterial population. In addition, Wanapat et al [51] concluded that phytonutrient pellets bind to cell membranes and disrupt ion exchanges, resulting in cell lysis and membrane breakdown. The supplementation of CMLP containing CT contributes both directly and indirectly to gram-positive bacteria, protozoa, and methanogens. Bioactive substances in feeds, whether in their natural form or as plant extracts, demonstrated the ability to influence the rumen’s capacity to decrease methane production by rumen microbes. Totakul et al [52] found that the addition of supplementary chaya pellets including 23.6% CP in DM, significantly increased bacterial cell counts compared to the control group. Additionally, Phesatcha et al [39] concluded that the addition of *Mitragyna* leaf pellets into the diet impacted protozoal populations and methanogenic archaea in Thai native beef cattle.

### Microbial protein synthesis

Table 4 presents data on urinary purine derivatives and other related indicators. Urinary purine derivatives and the excretion of allantoin were unchanged among all treatments (p> 0.05). The addition of CMLP increased the absorption of allantoin (p<0.05). The efficiency of microbial nitrogen synthesis (EMNS) and microbial nitrogen synthesis (MNS) significantly increased at higher levels of CMLP supplementation, particularly at 150 g (p<0.05). The highest microbial protein synthesis, as well as quantity and effectiveness, occurred at higher CMLP supplementation. This was attributed to the higher protein levels and CT ability of CMLP which improved rumen by-pass protein. The rumen microorganisms are an important source of protein, and microbial growth can influence the availability of amino acids. Consequently, increased CMLP supplementation proved more efficacious in microbial protein production. Essentially, Firkins et al [53] demonstrated that the synthesis of rumen microbial proteins enhanced the supply of proteins to the small intestine of ruminants. Moreover, CMLP supplementation significantly increased the levels of MNS and EMNS. Similarly, Phesatcha et al [45] indicated that *Flemingia macrophylla* pellets at a dosage of 150 g per day significantly enhanced nitrogen absorption and retention in lactating dairy cows. CMLP contains proteins and phytonutrients. Avila et al [13] reported that providing dairy cows with mangosteen peel rich in CT and SP promoted microbial protein synthesis efficiency, while reducing protozoal populations, improving dietary nitrogen absorption, and increasing microbial protein flow in the intestine. Additionally, beef cattle given lemongrass had lower EMNS according to Wanapat et al [16]. Variations in the effects on EMNS and urinary purine derivatives depended on the herb and dosage of the supplement, respectively.

## CONCLUSION

Our results suggested that CMLP supplementation, particularly at 150 g/h/d, enhanced nutrient digestibility, increased the propionic acid proportion, and promoted microbial protein synthesis while reducing protozoal populations and methane production. CMLP showed promise as an effective dietary protein supplement that improved rumen fermentation and performance of Thai native beef cattle.

## Figures and Tables

**Table 1:** Ingredients and chemical composition of the feeds used in the experiment

Items	Concentrate	Rice straw	CMLP
Feed ingredients (% of dry matter)			
Cassava chip	60.0		
Coconut meal	10.0		
Palm meal	18.0		
Rice bran	6.0		
Urea	2.0		
Salt	1.0		
Sulfur	0.5		
Mineral mixed	0.5		
Molasses	2.0		1.0
Cricket meal	-		50.0
Mangosteen peel powder	-		20.0
Lemongrass powder	-		20.0
Cassava starch	-		9.0
Chemical composition (% of dry matter)	92.5	93.0	95.8
Organic matter	92.7	91.5	94.1
Ash	7.3	8.5	5.9
Crude protein	14.7	2.3	38.5
Neutral detergent fiber	28.3	75.5	27.2
Acid detergent fiber	15.1	47.4	18.7
Condensed tannins	-	-	8.6

CMLP, pellet from cricket meal mixed phytonutrient containing mangosteen peel powder and lemongrass powder.

**Table 2:** Effects of insect protein and phytonutrient based tropical plant supplementation on voluntary feed intake and nutrient digestibility in beef cattle

Items	CMLP supplementation (g/h/d)				SEM	Contrasts		
	
	0	50	100	150		Linear	Quadratic	Cubic
Rice straw intake								
Kg of DM/d	2.7	3.2	3.6	3.6	0.39	0.06	0.56	0.38
Concentrate intake								
Kg of DM/d	2	2	2	2	-	-	-	-
CMLP								
Kg of DM/d	0	0.05	0.1	0.15	-	-	-	-
Total feed intake								
Kg of DM/d	4.7	5.2	5.7	5.8	0.39	0.06	0.56	0.38
Apparent digestibility (%)								
Dry matter	63.2	64.5	67.2	68.3	4.14	0.16	0.61	0.91
Organic matter	73.3	72.3	73.0	74.6	2.95	0.50	0.39	0.91
Crude protein	51.0^a^	52.8^b^	55.0^c^	57.1^d^	2.36	0.03	0.63	0.79
Neutral detergent fiber	59.2^a^	58.4^b^	56.1^c^	52.2^d^	3.91	0.04	0.46	0.99
Acid detergent fiber	48.1^a^	47.5^b^	45.1^c^	42.2^d^	3.12	0.02	0.46	0.90

a–d Means in the same row with different superscripts differed (p<0.05).

CMLP, pellet from cricket meal mixed phytonutrient containing mangosteen peel powder and lemongrass powder; SEM, standard error of the means; DM, dry matter.

**Table 3:** Effects of insect protein and phytonutrient based tropical plant supplementation on fermentation characteristics, blood urea nitrogen and microbial population in beef cattle

Items	CMLP supplementation (g/h/d)				SEM	Contrasts		
	
	0	50	100	150		Linear	Quadratic	Cubic
Ruminal pH	6.6	6.6	6.8	6.5	0.20	0.77	0.10	0.65
Ruminal temperature (°C)	38.7	38.0	38.1	38.2	0.61	0.24	0.06	0.76
NH_3_-N concentration (mg/dL)	17.0	16.0	16.4	15.2	1.84	0.07	0.63	0.57
Blood urea nitrogen (mg/dL)	12.5	12.1	11.3	11.0	0.94	0.82	1.00	0.32
Total VFA (mmol/L)	101.8	104.7	104.5	104.4	9.55	0.49	0.53	0.91
VFA productions (mol/100 mol)								
Acetic acid (C_2_)	76.5^a^	71.8^b^	71.9^b^	70.3^c^	2.84	0.04	0.78	0.85
Propionic acid (C_3_)	16.7^a^	19.6^b^	21.0^c^	22.6^d^	2.46	0.02	0.80	0.43
Butyric acid (C_4_)	6.9	8.6	7.1	7.3	2.47	0.46	0.89	0.58
Acetic/propionic acid ratio	4.6	3.7	3.7	3.1	0.71	0.06	0.71	0.50
Methane production (mM/L)^1)^	32.6^a^	30.4^b^	29.4^c^	28.3^d^	1.73	0.01	0.86	0.44
Rumen microbe population (cell/mL)								
Bacteria (×10^9^)	16.3^a^	17.0^b^	18.4^c^	18.9^c^	0.79	0.01	0.55	0.64
Protozoa (×10^6^)	6.2^a^	4.3^b^	4.3^b^	3.0^c^	0.86	0.01	0.42	0.14

1) Methane production = 0.45 (C_2_)–0.275 (C_3_)+0.4 (C_4_) calculated according to Moss et al [27].

a–d Means in the same row with different superscripts differed (p<0.05).

CMLP, pellet from cricket meal mixed phytonutrient containing mangosteen peel powder and lemongrass powder; SEM, standard error of the means; VFA, volatile fatty acids.

**Table 4:** Effects of insect protein and phytonutrient based tropical plant supplementation on urinary purine derivatives (PD) and microbial protein synthesis in beef cattle

Items	CMLP supplementation (g/h/d)				SEM	Contrasts		
	
	0	50	100	150		Linear	Quadratic	Cubic
Urinary purine derivatives (mmol/d)								
Allantoin excretion	51.4	54.5	57.4	59.9	1.43	0.85	0.17	0.56
Allantoin absorption	65.3^a^	67.7^b^	66.6^b^	73.7^c^	2.59	0.04	0.58	0.72
Microbial nitrogen supply (gN/d)	32.4^a^	35.3^b^	37.8^b^	41.2^c^	0.42	<0.01	0.06	0.34
EMNS (g/kg OMDR)^1)^	18.7^a^	22.2^b^	23.1^b^	25.1^b^	1.21	<0.01	0.06	0.26

1) OMDR, digestible organic matter apparently fermented in the rumen.

a–c Means in the same row with different superscripts differed (p<0.05).

CMLP, pellet from cricket meal mixed phytonutrient containing mangosteen peel powder and lemongrass powder; SEM, standard error of the means; EMNS, efficiency of microbial nitrogen supply.
